# Erector Spinae Plane Block Versus Caudal Block in Children: A Systematic Review and Meta-Analysis

**DOI:** 10.7759/cureus.87584

**Published:** 2025-07-09

**Authors:** Alexa Chedid, Rawan Masarwa, Rodrigo Muscogliati, Khaled Younes, Aya Hassanieh, Dima Ezzedine, Leen Najem, Caren Safi, Zeina Najem, Neel Badhe, Krystel Malek, Elie Najjar

**Affiliations:** 1 Medical School, Gilbert and Rose-Marie Chagoury School of Medicine, Lebanese American University, Byblos, LBN; 2 Centre for Spinal Studies and Surgery, Queens Medical Centre, Nottingham University Hospitals National Health Service (NHS) Trust, Nottingham, GBR; 3 Medical School, Hull York Medical School, University of York, York, GBR; 4 Medical School, University of Cambridge, Cambridge, GBR

**Keywords:** caudal block, erector spinae plane block, pain management, pediatric, rescue analgesia

## Abstract

Erector spinae plane block (ESPB) and caudal epidural block (CEB) are two regional anaesthetic techniques used in paediatric surgical analgesia. While CEB is a well-established method, ESPB has gained increasing interest due to its potential for prolonged analgesia. This systematic review and meta-analysis compared the efficacy and safety of ESPB and CEB in paediatric patients undergoing surgery. Nine randomised controlled trials (n = 612) were included. The primary outcome was time to first rescue analgesia, with secondary outcomes including the Face, Legs, Activity, Cry, and Consolability (FLACC) pain scores and adverse events. A random-effects model was applied due to high heterogeneity (I² > 90%). ESPB significantly prolonged the time to first rescue analgesia compared to CB (standard mean difference (SMD) = 2.75, p < 0.0001). At 24 hours postoperatively, ESPB showed improved FLACC scores (SMD = 0.25, p = 0.03), while CEB provided better analgesia at one hour (SMD = 0.51, p = 0.04). However, a similar proportion of patients in the ESPB group required rescue analgesia when compared to the CEB group (70% vs. 60%, p = 0.62), raising questions about its clinical benefit despite the longer duration of effect. Both techniques demonstrated low and comparable complication rates. ESPB appears to be a safe and effective alternative to CEB, offering prolonged analgesia with similar safety outcomes. However, the clinical relevance of its extended duration is limited by the comparable need for rescue analgesia. Future large-scale, standardised studies are needed to clarify its role in paediatric anaesthesia.

## Introduction and background

The pursuit of optimal postoperative analgesia in paediatric surgery continues to drive innovation in regional anaesthesia. Effective pain control is essential for minimising opioid requirements and promoting enhanced recovery, particularly in children, who are more susceptible to opioid-related adverse effects such as respiratory depression and nausea [1]. Regional anaesthesia techniques are central to addressing these concerns, with caudal epidural block (CEB) traditionally considered the standard approach for infraumbilical paediatric surgery due to its relative simplicity, favourable safety profile, and well-documented efficacy [2,3].

CEB involves ultrasound- or landmark-guided injection of local anaesthetic into the caudal epidural space via the sacral hiatus. Anatomically, the block is performed between the sacral cornua and coccyx, allowing local anaesthetic to ascend and surround the sacral and lower lumbar nerve roots. This results in both somatic and visceral analgesia [4,5]. The duration of analgesia following a single-shot CEB ranges between six and 12 hours, influenced by the local anaesthetic agent and its concentration [6,7]. Despite its effectiveness, CEB may be limited in cases requiring extended postoperative analgesia.

More recently, the erector spinae plane block (ESPB), first described by Forero et al. [8], has emerged as a promising alternative. In this technique, local anaesthetic is deposited into the fascial plane deep to the erector spinae muscle and superficial to the transverse process of the vertebra, most commonly at the lumbar or sacral levels for paediatric applications. This has been theorised to allows for craniocaudal spread of anaesthetic agents, affecting multiple spinal nerves through both dorsal and ventral rami, providing broader dermatomal coverage [6,9]; however, the definitive anatomy of ESPB spread is still being elucidated.

The technical execution of ESPB requires ultrasound guidance and is highly dependent on operator experience. The variability in technique, including the vertebral level (lumbar vs. sacral), volume and concentration of local anaesthetic, and patient anatomy, may contribute to inconsistencies in block efficacy [10,11]. Such heterogeneity necessitates standardisation of practice and critical appraisal of the current literature.

Pain assessment in young children, particularly those who cannot verbally report discomfort, relies on validated behavioural tools. The Face, Legs, Activity, Cry, and Consolability (FLACC) scale is one of the most widely adopted instruments for assessing procedural and postoperative pain in children aged two months to seven years. Each of the five behavioural components is scored from 0 to 2, yielding a total score from 0 (no pain) to 10 (severe pain), allowing clinicians to monitor pain intensity and response to intervention [12,13].

Although several studies have investigated the utility of ESPB in paediatric surgery, few have directly compared its performance with CEB. Findings remain mixed: while some trials report longer time to first rescue analgesia with ESPB, others paradoxically observe a higher overall requirement for rescue analgesia among patients receiving ESPB [7,14]. These discrepancies underscore the need for a systematic and rigorous comparison.

This systematic review and meta-analysis therefore aims to synthesise the current evidence comparing the efficacy, safety, and clinical utility of erector spinae plane block and caudal block in paediatric populations undergoing surgery. It seeks to clarify whether ESPB represents a reliable and advantageous alternative to caudal block in this vulnerable patient group.

## Review

Methods

Search Strategy

**Figure 1:**
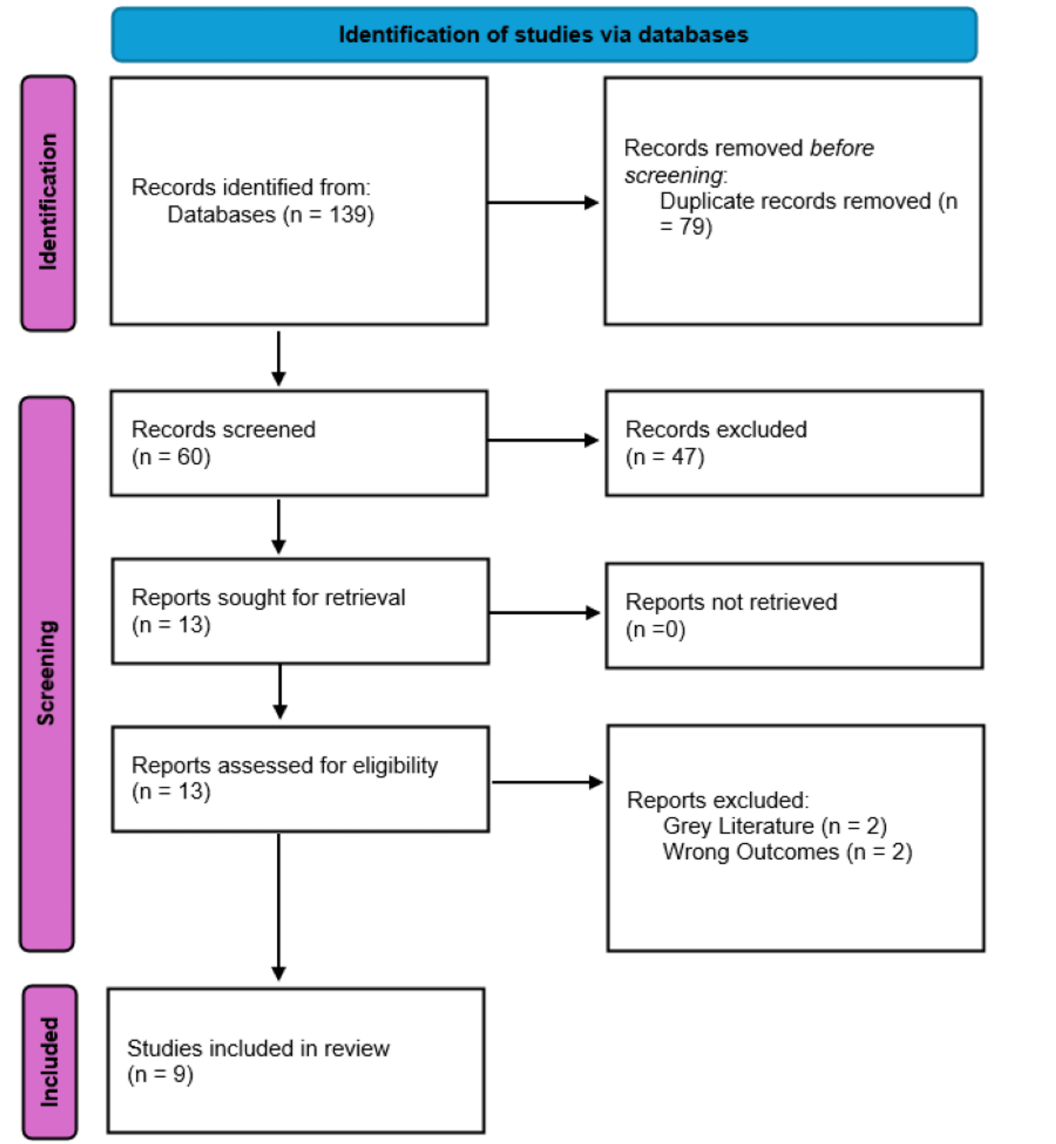
PRISMA flow diagram for systematic review. PRISMA: Preferred Reporting Items for Systematic Reviews and Meta-Analyses.

This systematic review followed Preferred Reporting Items for Systematic Reviews and Meta-Analyses (PRISMA) guidelines [15] to ensure comprehensive and transparent reporting (Figure 1). Searches were conducted across PubMed (MEDLINE), Web of Science, OVID (including EMBASE and APA PsycInfo), and Scopus, covering publications up to October 16, 2024. No financial support was received for this study, and authors declared no conflicts of interest.

The search terms were based on the population, intervention, comparison, and outcomes (PICO) framework (Table 1), focusing on combinations of keywords such as “caudal epidural block,” “caudal block,” “erector spinae block,” “sacral erector spinae block,” “erector spinae plane block,” and “ESPB.”

**Table 1:** PICO framework displaying the population, intervention, comparison, and outcomes of interest to this systematic review. PICO: population, intervention, comparison, and outcomes.

Population	Paediatric patients undergoing abdominal, lower limb, renal, and hypospadias repair surgeries
Intervention	Erector spinae plane block (ESPB)
Comparison	Caudal epidural block (CEB)
Outcomes	Postoperative pain reduction effectiveness (ESPB vs. CEB); pre- and post-operative opioid consumption (ESPB vs. CEB)

Gray Literature and Abstracts

Gray literature, clinical trial registries, and conference abstracts were excluded. Only peer-reviewed, full-text randomised controlled trials (RCTs) were included to ensure methodological rigour and relevance.

Inclusion and Exclusion Criteria

Randomised controlled trials directly comparing the analgesic efficacy of ESPB and CEB in paediatric surgeries were included. Studies were excluded if they did not specifically compare ESPB and CEB or focused solely on block mechanisms or non-comparative anaesthesia types, such as neonatal intensive care unit (NICU) protocols. Case series, non-paediatric studies, and research without comparative data were omitted. Prior to screening, duplicate articles were removed, and the remaining articles were screened based on their titles, abstracts, and full texts.

Data Extraction

Data extraction was conducted independently by eight researchers, with any disagreements resolved through consensus with a supervising researcher, ensuring consistency in the collected data. Collected data included study characteristics (title, year, design, authors, and country), patient demographics (sample size, age, sex ratio, and weight), and intervention details (block types, analgesic consumption, complications, block success rate, and hospital stay).

Statistical Analysis

The analysis employed RevMan 5.3 version software (Nordic Cochrane Centre, The Cochrane Collaboration, Copenhagen, 2014), using standardised mean difference for continuous outcomes, such as FLACC scores and time to rescue analgesia, and risk ratios for categorical outcomes, such as need for rescue analgesia and complication incidence. Heterogeneity was assessed via the I² statistic, and pooled analysis was performed using random-effects models due to the extent of substantial heterogeneity between studies. For outcomes with considerable heterogeneity (I^2^ > 75%) [16], subgroup analyses were performed to explore differences in surgical procedures that contributed to the observed variability.

Results

Study Selection and Risk of Bias Assessment

Of the 139 initial studies identified, nine randomised controlled trials met the inclusion criteria and were analysed to assess the comparative effectiveness of ESPB and CEB in paediatric postoperative analgesia. Key findings from these studies are outlined below [3,7,10,11,14,16-20].

The methodological quality of the nine included randomised controlled trials was assessed using the JBI Critical Appraisal Checklist for RCTs [21], with results being depicted in Table 2. All studies used randomisation and reported comparable baseline characteristics. However, allocation concealment was clearly described in only five studies, with others reporting insufficient or unclear methods. Blinding was a common limitation: while outcome assessor blinding was reported in most trials, only two studies blinded both participants and treatment providers. Despite this, outcome measures were collected using validated tools (e.g., FLACC scale) and applied consistently across groups. All studies reported complete follow-up and appropriate trial designs. The most suitable statistical methods were used, and participants were analysed as randomised. Overall, the studies were of moderate to high quality, with the main risk of bias related to blinding and allocation concealment.

**Table 2:** Depiction of the quality assessment of each included paper. Questions from Barker et al. [21] were used to assess each study. Y: Yes, N: No, U: Unclear. Q1. Was true randomisation used for assignment of participants to treatment groups? Q2. Was allocation to groups concealed? Q3. Were treatment groups similar at the baseline? Q4. Were participants blind to treatment assignment? Q5. Were those delivering the treatment blind to treatment assignment? Q6. Were treatment groups treated identically other than the intervention of interest? Q7. Were outcome assessors blind to treatment assignment? Q8. Were outcomes measured in the same way for treatment groups? Q9. Were outcomes measured in a reliable way? Q10. Was follow-up complete and, if not, were differences between groups in terms of their follow-up adequately described and analysed? Q11. Were participants analysed in the groups to which they were randomised? Q12. Was appropriate statistical analysis used? Q13. Was the trial design appropriate and any deviations from the standard RCT design (individual randomisation, parallel groups) accounted for in the conduct and analysis of the trial?

Study	Q1	Q2	Q3	Q4	Q5	Q6	Q7	Q8	Q9	Q10	Q11	Q12	Q13
Bansal et al. [11]	Y	Y	Y	N	N	Y	Y	Y	U	Y	Y	Y	Y
Pandey et al. [14]	Y	U	Y	U	N	Y	Y	Y	U	Y	Y	Y	Y
Guan et al. [7]	Y	U	Y	N	N	Y	Y	Y	U	Y	Y	Y	Y
Elshazly et al. [3]	Y	U	Y	N	N	U	Y	Y	U	Y	Y	Y	Y
Ozen et al. [20]	Y	Y	Y	Y	Y	Y	Y	Y	Y	Y	Y	Y	Y
Abdelrazik et al. [17]	Y	Y	Y	N	N	Y	Y	Y	U	Y	Y	Y	Y
Mandour et al. [19]	Y	Y	Y	Y	Y	Y	Y	Y	Y	Y	Y	Y	Y
Elbadry et al. [10]	Y	U	Y	N	N	Y	Y	Y	U	Y	Y	Y	Y
Abotaleb et al. [18]	Y	Y	Y	N	N	Y	Y	Y	U	Y	Y	Y	Y

Study Characteristics

Patient characteristics: The ESPB group included 305 patients, while 307 received CEB. Demographics such as age and weight did not differ significantly (mean age: 4.54 vs. 4.29 years, p = 0.53; mean weight: 18.9 kg vs. 18.24 kg, p = 0.55). Gender distribution was predominantly male (85%), which reflects the nature of the included procedures, such as hypospadias repair, circumcision, and inguinal hernia, and does not imply any clinically meaningful difference between groups.

Surgical Characteristics

The mean duration of surgery was comparable between the ESPB and CEB groups, with ESPB averaging 75.28 minutes and CEB 74.11 minutes, showing no statistically significant difference (unpaired t-test, p = 0.95). Both block techniques were applied across a variety of paediatric surgical procedures, spanning abdominal, urological, and orthopaedic categories.

Abdominal surgeries: Elective abdominal procedures included inguinal hernia repair, hydrocele repair, and appendectomy.

Urological surgeries: Hypospadias repair was performed in a cohort of 50 patients, along with other procedures, including nephrectomy and pyeloplasty.

Orthopaedic surgeries: Paediatric lower limb procedures consisted of femoral and tibial shaft fracture repairs, as well as corrections for genu varum and genu valgum. Hip and proximal femur surgeries were also included.

The diversity of procedures further supports the comparable applicability and utility of ESPB and CEB in managing postoperative analgesia across a broad spectrum of paediatric surgeries. Table 3 shows further details on study characteristics and demographics.

**Table 3:** General characteristics of the included literature. ESPB: erector spinae plane block; CEB: caudal block, PB: penile block; SD: standard deviation; IQR: inter-quartile range.

Author (year)	Sample size, n	Age in years: SD (IQR)	Type of surgery	Surgery duration in minutes: SD (IQR)
Bansal et al. (2024) [11]	Total: 50; ESPB: 25; CEB: 25	Mean ESPB: 4.76 (1.69); Mean CEB: 4.96 (1.59)	Hypospadias repair.	Mean ESPB: 57.0 (3.23); Mean CEB: 55.8 (4.25)
Pandey et al. (2024) [14]	Total: 52; ESPB: 26; CEB: 26	Median ESPB: 5.0 (3.0, 6.0); Median CEB: 4.5 (2.0, 6.0)	Elective abdominal surgery.	Median ESPB: 120 (60-180); Median CEB: 120 (60-120)
Guan et al. (2023) [7]	Total: 98; ESPB: 32; CEB: 33; Control: 33	Median ESPB: 3.3 (2.9–3.8); Median CEB: 3.4 (3.0–3.8); Median Control: 3.2 (2.8–4.0)	Unilateral open inguinal hernia repair.	Median ESPB: 25 (22-29); Median CEB: 25 (24-29); Median control: 24 (21-27)
Elshazly et al. (2023) [3]	Total: 76; ESPB: 38; CEB: 38	Mean ESPB: 4.57 (2.99); Mean CEB: 4.16 (3.76)	Paediatric hip and proximal femur surgery.	Mean ESPB: 62 (18); Mean CEB: 78 (24)
Ozen et al. (2024) [20]	Total: 150; ESPB: 75; CEB: 75	Median ESPB: 4 (3.75–5.25); Median CEB: 4 (3–5)	Paediatric circumcision.	Median ESPB: 39.50 (38–41); Median CEB: 38 (34–41.25)
Abdelrazik et al. (2022) [17]	Total: 60; ESPB: 20; CEB: 20; Control: 20	Mean ESPB: 3.85 (1.53); Mean CEB: 3.58 (1.48); Mean control: 3.90 (1.59)	Inguinal hernia repair; hydrocele repair; appendectomy.	Mean ESPB: 28.3 (3.4); Mean CEB: 27.6 (4.5); Control: 28.7 (3.7)
Mandour et al. (2023) [19]	Total: 50; ESPB: 25; CEB: 25	Mean ESPB: 4.56 (1.69); Mean CEB: 4.40 (1.73)	Nephrectomies; pyeloplasties.	Mean ESPB: 93 (19); Mean CEB: 88 (19)
ElBadry et al. (2023) [10]	Total: 120; ESPB: 39; CEB: 40; Penile block: 41	Mean ESPB: 2.85 (1.29); Mean CEB: 2.75 (1.35); Mean PB: 2.95 (1.07)	Hypospadias repair.	ESPB: 118 (36); Mean CEB: 116 (33); Mean PB: 112 (34)
Abotaleb et al. (2023) [18]	Total: 50; ESPB: 25; CEB: 25	Mean ESPB: 7.96 (3.55); Mean CEB: 7.36 (3.49)	Femoral and tibial percutaneous fixation with gliding nails; high tibial and distal femur osteotomy.	Mean ESPB: 134 (26); Mean CEB: 138 (26)

Primary Outcomes

**Figure 2:**
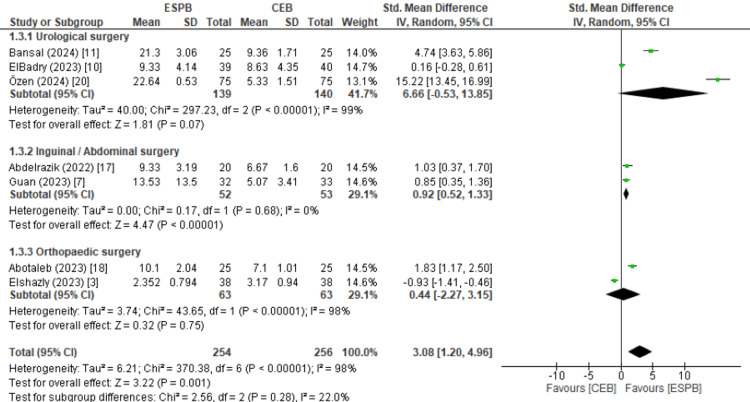
Forest plot showing the meta-analysis calculation for time to first rescue analgesia, with subgroups for urological, inguinal/abdominal, and orthopaedic surgical procedures. ESPB: erector spinae plane block; CEB: caudal block.

The time to first rescue analgesia, a key marker of prolonged analgesic efficacy, was significantly longer for ESPB than CEB (standard mean difference (SMD): 3.08, 95% CI: 1.20-4.96; 13.2 hours vs. 6.27 hours; p < 0.001), as shown in Figure 2. This finding varied depending on the type of surgery, with inguinal and abdominal surgeries showing more consistent results favouring ESPB, while urological and surgical procedures showed more variability. However, the I^2^ value for time to first rescue analgesia when looking at all surgical procedures was 98%, indicating considerable heterogeneity between studies.

While 70% of patients in the ESPB group required rescue analgesia compared to 60% in the CEB group, this difference was not statistically significant (relative risk (RR): 1.12, 95% CI: 0.72-1.73; p = 0.62), indicating that extended analgesia in ESPB did not substantially impact rescue analgesia requirements.

Secondary Outcomes

**Figure 3:**
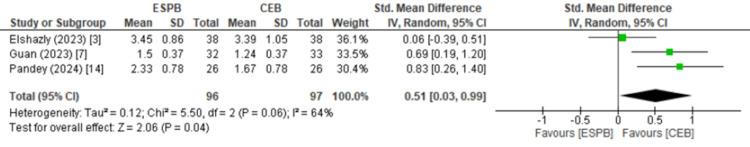
Forest plot showing the meta-analysis calculation for the FLACC score at one hour. FLACC: Face, Legs, Activity, Cry, and Consolability; ESPB: erector spinae plane block; CEB: caudal block.

**Figure 4:**
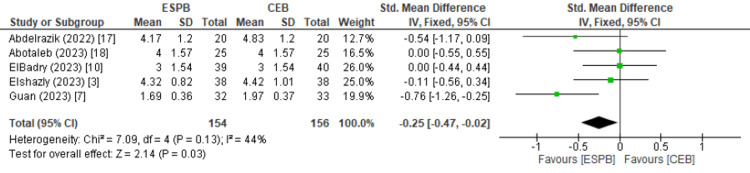
Forest plot showing the meta-analysis calculation for the FLACC score at 24 hours. FLACC: Face, Legs, Activity, Cry, and Consolability; ESPB: erector spinae plane block; CEB: caudal block.

Pain control: FLACC scores indicated slightly worse pain control with ESPB at one hour (1.82 vs. 1.70; SMD: 0.51, 95% CI: 0.03-0.99; p = 0.04), but better pain control with ESPB at 24 hours (3.19 vs. 3.31; SMD: -0.25, 95% CI: -0.47 to -0.02; p = 0.03). However, these findings were associated with moderate heterogeneity (I² = 55% at 1 hour; I² = 48% at 24 hours). Additionally, these absolute differences (0.12 points) are not clinically meaningful, as differences less than 0.5 on the FLACC scale are generally considered negligible. Therefore, the statistical significance should be interpreted cautiously and not assumed to indicate superior clinical efficacy. No significant differences were found at two, six, or 12 hours (p = 0.49, 0.07, and 0.26), respectively (Figures 3, 4).

Adverse events: Complication rates were low and comparable, with CEB reporting 11.5% and ESPB 8.6% (RR: 0.78, 95% CI: 0.41-1.47; p = 0.44).

Discussion

Effective postoperative pain management is crucial, especially in paediatric populations where untreated pain can adversely affect immediate recovery and quality of life [21,22]. Despite this, paediatric pain management is frequently overlooked. To our knowledge, this is the first systematic review focused exclusively on comparing ESPB and CEB outcomes in paediatric patients, filling a gap in the literature given the rising interest in ESPB as an alternative to CEB for prolonged analgesia. Among the nine randomised controlled trials meeting our inclusion criteria, our analysis suggests that while CEB offers better analgesia immediately after surgery, ESPB offers more prolonged pain control.

Postoperative Outcomes

Across the included studies, ESPB consistently provided a significantly longer duration of analgesia than CEB, with an average time to first rescue analgesia of 13.2 hours compared to 6.27 hours in the CEB group, as shown in Figure 2. This prolonged analgesic effect suggests that ESPB supports an extended pain-free period following surgery, which is particularly advantageous in procedures such as lower abdominal and lower limb surgeries [23,24]. Notably, this effect has been observed across a wide range of paediatric surgeries included in our review, from urological procedures like hypospadias repair to orthopaedic interventions involving femoral and tibial fractures, as well as abdominal surgeries such as inguinal hernia and appendectomy [3,7,10,11,14,17-20].

ESPB’s extended analgesic effect across diverse surgical types reinforces its utility as a versatile tool in paediatric anaesthesia. Abdelrazik et al. [17], for example, documented an extended time to first rescue analgesia in paediatric patients undergoing lower abdominal surgeries with ESPB versus CEB, a result echoed in similar studies within different surgical contexts [7,11,18,20]. This repeatability across varied patient demographics, ages, and surgical types highlights ESPB’s potential as a viable method for achieving postoperative pain control in paediatric patients, regardless of the specific type of surgery. The extended pain relief period provided by ESPB could support improved postoperative recovery and patient comfort, particularly in surgeries where prolonged analgesia is beneficial [24].

However, despite ESPB’s longer analgesic duration, the overall percentage of patients requiring rescue analgesia did not differ significantly between groups (70% in ESPB vs. 60% in CEB, p = 0.62). This finding suggests that while ESPB effectively delays the onset of postoperative pain, it may not reduce the overall need for additional analgesia compared to CEB. The clinical value of this delay is therefore limited unless tied to functional outcomes such as earlier ambulation or reduced opioid consumption [25].

Pain Control (FLACC)

Pain control, assessed using the FLACC score, was superior with CEB in the immediate postoperative period, but ESPB provided more effective prolonged analgesia. At one hour post-surgery, CEB was associated with significantly lower FLACC scores compared to ESPB (mean 1.70 vs. 1.82; SMD = 0.51; p = 0.04), indicating better early pain relief. However, by 24 hours postoperatively, ESPB demonstrated improved FLACC scores over CEB (mean 3.19 vs. 3.31; SMD = 0.25; p = 0.03), suggesting more sustained analgesic benefit. Guan et al. [7] also reported lower FLACC scores after surgery with ESPB, hypothesising that the longer analgesic duration may be due to the anatomical distribution of anaesthetics in ESPB. While the anatomy of ESPB spread is still being elucidated, several mechanisms of action have been hypothesised. This includes analgesic spread through the erector spinae muscle plane to the dorsal and ventral rami, as well as anaesthetic spread to the epidural and paravertebral spaces [26,27].

However, even though there were statistically significant differences in FLACC scores between ESPB and CEB at one hour and 24 hours after surgery, this does not directly translate to clinical significance. A difference of at least >0.5 in FLACC scores is typically considered clinically significant, meaning that CEB is clinically better than ESPB at one hour after surgery (SMD = 0.51). But the difference in ESPB to CEB at 24 hours after surgery is small (SMD = 0.25), and unlikely to significantly impact patients in clinical practice.

Additionally, results from Pandey et al. [14], despite also showing a lower FLACC score in the ESPB group when compared to the CEB group, still noted that CEB provided longer and better-quality analgesia when compared to ESPB. This finding directly challenges the broader trend identified in our review and highlights the need for caution in interpreting ESPB as superior in proving prolonged analgesia. Rather than favouring one technique universally, these findings underscore the importance of continued research into ESPB’s efficacy across different surgical applications and anatomical sites to better understand its utility as a postoperative pain management technique in paediatrics.

Complications of the Surgery

The studies reviewed indicated a low overall incidence of complications related to analgesia, with ESPB and CEB showing comparable safety profiles (p = 0.44). Across studies, both blocks appeared generally safe, with adverse effects occurring infrequently and without significant differences between groups. Abdelrazik et al. [17] observed urinary retention in two patients within the CEB group, as well as similar rates of nausea and vomiting in both ESPB and CEB patients, suggesting no marked safety advantage of one technique over the other. ElBadry et al. [10] reported additional adverse effects, including penile engorgement, bradycardia, hypotension, and vomiting, which again showed no significant variation between the two groups.

However, given the relatively small sample size of this review, as well as short follow-up, it is inappropriate for this study to draw significant conclusions in terms of complications of ESPB and CEB, especially given the possibility of rare but serious complications that can occur with some forms of regional analgesia, such as epidural haematomas. It is possible that complications reported in this study are inherent features of regional forms of anaesthesia rather than the block techniques themselves [28]. Furthermore, the lack of detailed reporting on complications in several studies limits the ability to draw definitive conclusions regarding any minor differences in safety profiles. Given these findings, ESPB and CEB can both be considered safe options for paediatric analgesia in lower abdominal and lower limb surgeries, with a low risk of complications. Further studies with standardised reporting on adverse effects, larger patient populations and long-term follow-up could provide additional clarity regarding complications of ESPB and CEB.

Clinical Implications and Considerations

The findings from this review suggest that ESPB can serve as a versatile alternative to CEB in paediatric surgery, particularly where prolonged postoperative analgesia is desirable. ESPB may be valuable in settings lacking resources for general anaesthesia follow-up, as its prolonged duration could reduce reliance on supplemental opioids. However, the variation in ESPB technique and the need for ultrasound guidance highlight the importance of training and operator skill. Implementation in low-resource settings may be constrained by access to ultrasound and trained anaesthetists.

From a cost-effectiveness perspective, although ESPB may reduce postoperative opioid use and related side effects, the cost and training required for ultrasound-guided administration should be factored into implementation strategies. Future studies should include economic analyses comparing ESPB and CEB in various healthcare settings.

Limitations

While this systematic review of RCTs provides a high level of evidence, certain limitations warrant consideration. The total number of patients across studies was relatively small, which may affect the statistical power of our findings. Although RCTs are designed to minimise bias and provide robust comparative data, the limited sample sizes highlight the need for larger-scale studies to confirm the observed trends.

Additionally, there was significant statistical and clinical heterogeneity in the included studies, which could have influenced the interpretation of pooled results. The primary outcome, time to first rescue analgesia, showed substantial heterogeneity (I² = 98%), and similar inconsistency was observed in secondary outcomes such as FLACC scores. As a result, to mitigate the effect of heterogeneity, a random-effects model was applied in the meta-analysis to account for between-study differences and perform a subgroup analysis based on the surgical procedures of each study, as shown in Figure 2. However, these approaches do not fully eliminate the impact of heterogeneity on result interpretation; thus, the findings of this systematic review need to be interpreted with caution. This statistical heterogeneity likely reflects variability in surgical types and patient demographics across studies. For instance, inguinal and abdominal surgeries showed significantly longer times to first rescue analgesia with ESPB; however, the same was not seen with orthopaedic and urological procedures, as shown in Figure 2. Differences in surgical procedures, anaesthetic dosages, and patient characteristics may influence the efficacy and safety outcomes observed for ESPB and CEB. Future studies should aim to standardise these variables to better clarify ESPB’s role in paediatric postoperative pain management and to develop consistent protocols for its use in clinical practice.

A further limitation is the substantial variation in ESPB technique between studies. Approaches ranged from lumbar to sacral level, and differed in terms of injection volume and local anaesthetic concentration. These inconsistencies make direct comparison across trials challenging and may contribute to outcome variability. Moreover, the successful performance of ESPB is dependent on operator skill and familiarity with the procedure. The learning curve associated with this technique, as well as variations in practitioner experience across centres, could have influenced block efficacy and safety outcomes. Future studies should aim to standardise ESPB protocols and account for operator training to ensure reproducibility and clinical applicability.

## Conclusions

This systematic review highlights that while ESPB may offer a longer time to first rescue analgesia and slightly improved pain scores at certain postoperative intervals compared to CEB, these results must be interpreted with caution. Notably, CEB provided better analgesia at one hour after surgery, suggesting superior early postoperative pain control. Importantly, the overall proportion of patients requiring rescue analgesia was not significantly reduced in the ESPB group, raising questions about the clinical relevance of its extended duration. Moreover, there were inconsistencies in findings, with one included study reporting that CEB provided longer and higher-quality analgesia when compared to ESPB, directly challenging the general trend observed across other trials.

Both ESPB and CEB demonstrated acceptable safety profiles and remain viable options for paediatric postoperative pain control. However, given the variability in ESPB technique and the inconsistencies in patient outcomes, it is premature to conclude that ESPB is categorically superior. Clinical decision-making should prioritise outcomes that are meaningful to patients, such as reduced opioid consumption, improved comfort, and earlier discharge. To validate these findings and develop standardised clinical protocols, further high-quality studies with larger sample sizes, longer follow-up periods, and consistent block techniques are essential.
